# Supplementary material for: Valorization of Common (Fagopyrum esculentum Moench.) and Tartary (Fagopyrum tataricum Gaertn.) Buckwheat in Gluten-Free Polenta Samples: Chemical-Physical and Sensory Characterization
Source: Foods. 2022 Oct 30;11(21):3442. doi: 10.3390/foods11213442 (PMC9656078; doi:10.3390/foods11213442)
Supplement: Supplementary file 1 [file foods-11-03442-s001.zip › Supplementary material_Figure S1.pdf]

Supplementary Figure S1. Mean values for the interactions Judges × Replicates (J×R), Judges × Product (J×P), and Product × Replicates (P×R) for the sensory attributes:

a) Ochre color

b) Bitter taste

c) Astringency

d) Gumminess

e) Moisture

f) Firmness

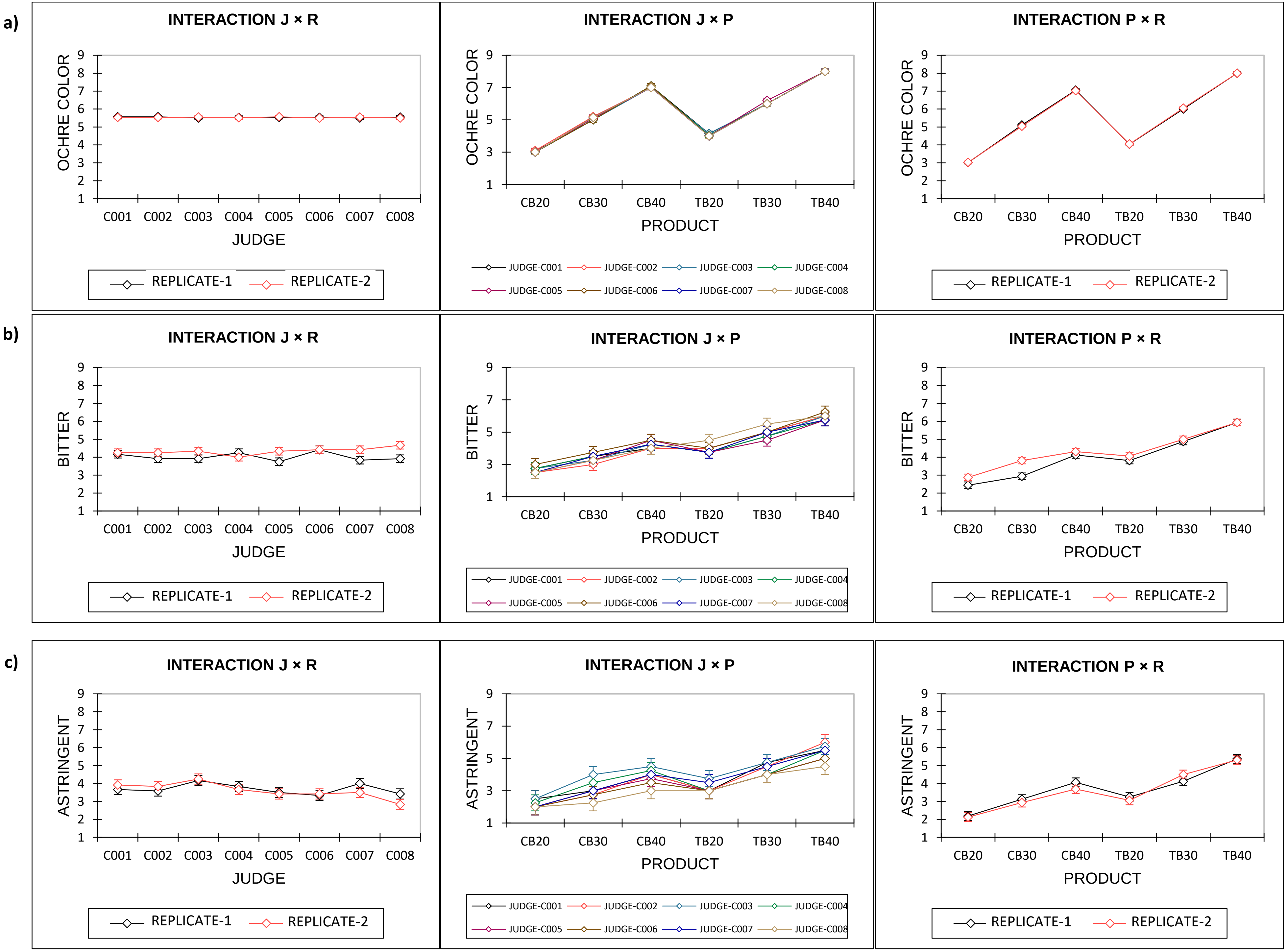
a)
INTERACTION J × R
OCHRE COLOR
JUDGE
C001 C002 C003 C004 C005 C006 C007 C008
REPLICATE-1 REPLICATE-2
INTERACTION J × P
OCHRE COLOR
PRODUCT
CB20 CB30 CB40 TB20 TB30 TB40
JUDGE-C001 JUDGE-C002 JUDGE-C003 JUDGE-C004 JUDGE-C005 JUDGE-C006 JUDGE-C007 JUDGE-C008
INTERACTION P × R
OCHRE COLOR
PRODUCT
CB20 CB30 CB40 TB20 TB30 TB40
REPLICATE-1 REPLICATE-2
b)
INTERACTION J × R
BITTER
JUDGE
C001 C002 C003 C004 C005 C006 C007 C008
REPLICATE-1 REPLICATE-2
INTERACTION J × P
BITTER
PRODUCT
CB20 CB30 CB40 TB20 TB30 TB40
JUDGE-C001 JUDGE-C002 JUDGE-C003 JUDGE-C004 JUDGE-C005 JUDGE-C006 JUDGE-C007 JUDGE-C008
INTERACTION P × R
BITTER
PRODUCT
CB20 CB30 CB40 TB20 TB30 TB40
REPLICATE-1 REPLICATE-2
c)
INTERACTION J × R
ASTRINGENT
JUDGE
C001 C002 C003 C004 C005 C006 C007 C008
REPLICATE-1 REPLICATE-2
INTERACTION J × P
ASTRINGENT
PRODUCT
CB20 CB30 CB40 TB20 TB30 TB40
JUDGE-C001 JUDGE-C002 JUDGE-C003 JUDGE-C004 JUDGE-C005 JUDGE-C006 JUDGE-C007 JUDGE-C008
INTERACTION P × R
ASTRINGENT
PRODUCT
CB20 CB30 CB40 TB20 TB30 TB40
REPLICATE-1 REPLICATE-2

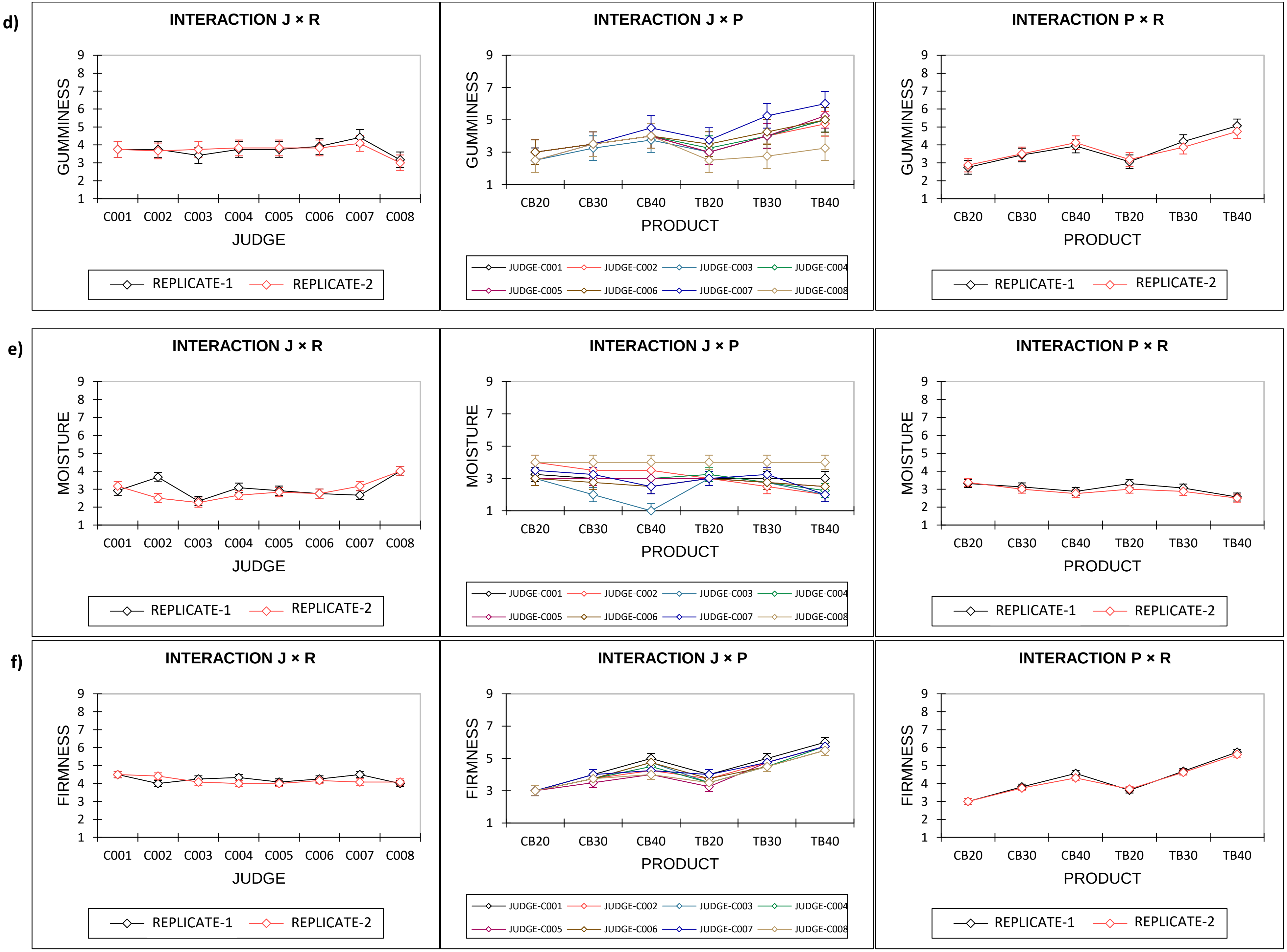
d)
INTERACTION J × R
GUMMINESS
9 8 7 6 5 4 3 2 1
C001 C002 C003 C004 C005 C006 C007 C008
JUDGE
REPLICATE-1 REPLICATE-2
INTERACTION J × P
GUMMINESS
9 7 5 3 1
CB20 CB30 CB40 TB20 TB30 TB40
PRODUCT
JUDGE-C001 JUDGE-C002 JUDGE-C003 JUDGE-C004
JUDGE-C005 JUDGE-C006 JUDGE-C007 JUDGE-C008
INTERACTION P × R
GUMMINESS
9 8 7 6 5 4 3 2 1
CB20 CB30 CB40 TB20 TB30 TB40
PRODUCT
REPLICATE-1 REPLICATE-2
e)
INTERACTION J × R
MOISTURE
9 8 7 6 5 4 3 2 1
C001 C002 C003 C004 C005 C006 C007 C008
JUDGE
REPLICATE-1 REPLICATE-2
INTERACTION J × P
MOISTURE
9 7 5 3 1
CB20 CB30 CB40 TB20 TB30 TB40
PRODUCT
JUDGE-C001 JUDGE-C002 JUDGE-C003 JUDGE-C004
JUDGE-C005 JUDGE-C006 JUDGE-C007 JUDGE-C008
INTERACTION P × R
MOISTURE
9 8 7 6 5 4 3 2 1
CB20 CB30 CB40 TB20 TB30 TB40
PRODUCT
REPLICATE-1 REPLICATE-2
f)
INTERACTION J × R
FIRMNESS
9 8 7 6 5 4 3 2 1
C001 C002 C003 C004 C005 C006 C007 C008
JUDGE
REPLICATE-1 REPLICATE-2
INTERACTION J × P
FIRMNESS
9 7 5 3 1
CB20 CB30 CB40 TB20 TB30 TB40
PRODUCT
JUDGE-C001 JUDGE-C002 JUDGE-C003 JUDGE-C004
JUDGE-C005 JUDGE-C006 JUDGE-C007 JUDGE-C008
INTERACTION P × R
FIRMNESS
9 8 7 6 5 4 3 2 1
CB20 CB30 CB40 TB20 TB30 TB40
PRODUCT
REPLICATE-1 REPLICATE-2
