# Supplementary material for: Valorization of Common (Fagopyrum esculentum Moench.) and Tartary (Fagopyrum tataricum Gaertn.) Buckwheat in Gluten-Free Polenta Samples: Chemical-Physical and Sensory Characterization
Source: Foods. 2022 Oct 30;11(21):3442. doi: 10.3390/foods11213442 (PMC9656078; doi:10.3390/foods11213442)
Supplement: Supplementary file 1 [file foods-11-03442-s001.zip › Supplementary material_Table S1.pdf]

Supplementary table S1. Attributes used for sensory profile with their definitions and reference standards.

Sensory Descriptor	Definition	Reference Standard
	APPEARANCE	
Ochre color	Characteristic perceived through the sense of sight referring to the yellow-ochre color	Ochre acrylic color (n° 14131) (Maimeri S.p.A, Mediglia, Italy)
	TASTE	
Bitter	One of the basic tastes caused by bitter compounds (e.g. caffeine) perceived in the oral cavity	0.27 g of caffeine monohydrate (Sigma Aldrich srl, Milano, Italy) in 500 mL of still mineral water (Levissima S.p.A.)
	TEXTURE	
Firmness	Force required to compress the sample between the molars.	Mozzarella cheese for pizza (biG, S.r.l, Milano, Italy)
Gumminess	Time required to disintegrate a semi-solid food to a state ready for swallowing	Scamorza Cheese (Esselunga, S.p.A, Limito di Pioltello, Italy)
Moisture	Perceived degree of humidity while chewing	Primo Sale cheese (Carrefour, GS S.p.A., Milano, Italy)
	TACTILE SENSATIONS	
Astringent	Tactile sensation of dryness and puckering perceived in the oral cavity	2.00 g of Potassium alum (Sigma Aldrich srl, Milano, Italy) in 500 mL of still mineral water (Levissima S.p.A.)
